# IgG anti-RBD levels during 8-month follow-up post-vaccination with BNT162b2 and mRNA-1273 vaccines in healthcare workers: A one-center study

**DOI:** 10.3389/fcimb.2022.1035155

**Published:** 2022-11-30

**Authors:** Sergio Gil-Manso, Roberto Alonso, Pilar Catalán, Ignacio Sánchez-Arcilla, Marco Marzola, Rafael Correa-Rocha, Marjorie Pion, Patricia Muñoz

**Affiliations:** ^1^ Advanced ImmunoRegulation Group, Instituto de Investigación Sanitaria Gregorio Marañón, Hospital General Universitario Gregorio Marañón, Madrid, Spain; ^2^ Department of Clinical Microbiology and Infectious Diseases, Hospital General Universitario Gregorio Marañón, Madrid, Spain; ^3^ Instituto de Investigación Sanitaria Gregorio Marañón, Hospital General Universitario Gregorio Marañón, Madrid, Spain; ^4^ CIBER (Centro de Investigación Biomédicas en Red) de Enfermedades Respiratorias, CIBERES, Barcelona, Spain; ^5^ Department of Medicine, Facultad de Medicina, Universidad Complutense de Madrid, Madrid, Spain; ^6^ Department of Labour Risks Prevention, Hospital General Universitario Gregorio Marañón, Madrid, Spain; ^7^ Laboratory of Immune-Regulation, Instituto de Investigación Sanitaria Gregorio Marañón, Hospital General Universitario Gregorio Marañón, Madrid, Spain

**Keywords:** COVID-19, mRNA vaccines, antibodies, humoral response, IgG Anti-S

## Abstract

**Introduction:**

Since the COVID-19 outbreak, specific mRNA-based anti-SARS-CoV-2 vaccines have been developed and distributed worldwide. Because this is the first time that mRNA vaccines have been used, there are several questions regarding their capacity to confer immunity and the durability of the specific anti-SARS-CoV-2 response. Therefore, the objective of this study was to recruit a large cohort of healthcare workers from the Gregorio Marañón Hospital vaccinated with the mRNA-1273 or BNT126b2 vaccines and to follow-up on IgG anti-RBD levels at 8 months post-vaccination.

**Methods:**

We recruited 4,970 volunteers and measured IgG anti-RBD antibodies on days 30 and 240 post-vaccination.

**Results:**

We observed that both vaccines induced high levels of antibodies on day 30, while a drastic wane was observed on day 240, where mRNA-1273 vaccinated induced higher levels than BNT162b2. Stratifying by vaccine type, age, gender, and comorbidities, we identified that older mRNA-1273-vaccinated volunteers had higher antibody levels than the younger volunteers, contrary to what was observed in the BNT162b2-vaccinated volunteers.

**Discussion:**

In conclusion, we observed that mRNA-1273 has a higher capacity to induce a humoral response than BNT162b2 and that age is a factor in the specific response.

## Introduction

Following the COVID-19 outbreak, by May 2022, more than 527 million cases of SARS-CoV-2 infection and more than 6.28 million associated deaths were reported. In Spain, 12.2 million cases and more than 100,000 deaths have been registered. Since the appearance of the first cases, several drugs, such as remdesivir (Beigel et al., 2020; Gottlieb et al., 2022), dexamethasone (Group et al., 2021), and tocilizumab (Mariette et al., 2021; Salama et al., 2021), among others, have been used as unspecific treatments for the disease. The majority of antiviral drugs designed to treat SARS-CoV-2 are in clinical trials. Thus far, only minor to moderate improvements in clinical outcome have been observed, and some of these drugs present non-negligible side effects. Therefore, vaccination is thought to be the only global solution to avoid viral spread and reduce the frequency of severe symptoms. Since the beginning of 2020, several vaccines have been developed and are currently in use (Barouch, 2022), many of which are based on mRNA technology (Vitiello and Ferrara, 2021). These vaccines rely on a specific mRNA sequence contained in a lipid nanoparticle that is introduced into host cells and in order to synthesize the protein codified by the specific sequence (Bettini and Locci, 2021). In the context of COVID-19, the two main vaccine candidates developed were the BNT162b2 (Pfizer/BioNTech) (Polack et al., 2020) and mRNA-1273 (Moderna) (Jackson et al., 2020) vaccines. The BNT162b2 vaccine is composed of the specific mRNA sequence for the spike protein of the SARS-CoV-2 original strain, ALC-0315, ALC-0159, DSPC, and cholesterol. In contrast, the mRNA-1273 vaccine comprises the mRNA sequence, SM-102, PEG-DMG, DSCP, and cholesterol (Schoenmaker et al., 2021).

These vaccines were approved by the European Medicines Agency (EMA) in Europe by the end of 2020 (Agency EM, 2021a; Agency EM, 2021b), and at-risk populations, including healthcare workers, started to be vaccinated by the beginning of 2021. Both mRNA vaccines required two doses for complete vaccination, separated by 21 days in the case of BNT162b2 and 28 days in the case of mRNA-1273. These vaccines have demonstrated efficacy in generating a humoral response, with high antibody levels being reached in the first weeks post-vaccination (Doria-Rose et al., 2021; Gil-Manso et al., 2021; Wheeler et al., 2021). Although these antibodies can be detected several months post-vaccination, a wane in antibody levels after 6 months has been observed (Naaber et al., 2021; Gil-Manso et al., 2022; Herzberg et al., 2022; Jalkanen et al., 2022). However, despite many studies concerning the humoral response, few compare the outcomes of vaccination in cohorts receiving BNT162b2 or mRNA-1273. Furthermore, these studies were carried out with a reduced number of individuals, limiting the robusticity of any conclusions drawn.

For these reasons, this study aimed to compare the generation and evolution of antibodies against the spike protein of SARS-CoV-2, after vaccination with two doses of the two mRNA-based vaccines in a large, well-controlled population of individuals to obtain robust conclusions regarding the humoral response.

## Methods

This study was approved by the research ethics committee of the Hospital General Universitario Gregorio Marañón (MICRO.HGUGM.2020-021) and was performed according to the principles of the Declaration of Helsinki and the European Union Regulation 2016/679. This hospital is one of the largest hospitals in Spain, serving a population of 750,000 inhabitants, with 1,091 functional hospitalization beds and 8,499 health workers in the year 2020 (Sanidad, 2020).

As soon as the first vaccines against SARS-CoV-2 based on mRNA technology were approved, they were offered to all the hospital’s health workers. First doses were administered starting on the 10th of January 2021, and second doses were given 21 days or 28 days after the first for BNT162b2 and mRNA-1273, respectively. By the 28th of February, 8,565 employees were fully vaccinated (there was an increase in the number of healthcare workers from 2020, but official numbers have not been published), of which 1,599 individuals received mRNA-1273 and 6,966 individuals received BNT162b2. Most health workers were vaccinated with BNT162b2 due to its higher availability at the time of vaccination compared to the mRNA-1273 vaccine. All vaccinated workers were offered an antibody quantification in March 2021 and October 2021, 30 and 240 days post-full vaccination, before the third boosting dose was offered, to check for long-term seropositivity.

The quantification of anti-SARS-CoV-2 IgG antibodies targeting the viral spike protein in serum samples was carried out using a quantitative chemiluminescent assay (SARS-CoV-2 IgG II Quant Reagent Kit) and an ARCHITECT i2000 instrument (Abbott; Chicago, USA). IgG levels were expressed in AU/mL (arbitrary units per millilitre) and were converted to BAU/mL (binding antibody units per millilitre) using the conversion coefficient provided by the manufacturer (1 BAU = 0.142 X AU) to standardize the results. The linear detection range was from 0 to 5,680 BAU/mL. Results above 7.10 BAU/mL were considered positive.

A statistical analysis was carried out and figures were created using GraphPad version 8.0 (GraphPad Software, Inc; California, USA). For multiple comparisons, a Kruskal Wallis test and Dunn’s test correction were used. A chi-squared test was used for categorical variables and t-tests were used for quantitative variables. Some analyses needed multiple variables when the difference between IgG levels was compared with the integration of age, gender, and/or comorbidities using the mvaghermite integration method. In the correlation analysis, a Spearman correlation test was used for all variables. A mixed-effects model was used to study the evolution and interaction of IgG Anti-RBD levels. The values were logarithmically transformed, and tobit regression was used.

## Results

### Vaccinated individual and group characteristics

8,565 health workers were fully vaccinated against COVID-19 with the mRNA vaccines BNT162b2 or mRNA-1273 during January and February 2021. While the first antibody quantification was performed in 6,219 workers (72.6% of the total workers vaccinated during this period), only 4,970 workers (58%) participated in both extractions. Of the 4,970 volunteers, 783 were vaccinated with the mRNA-1273 vaccine (15.75% of the total sample), and 4,187 were vaccinated with BNT162b2 (84.25%, **Table 1**).

**Table 1:** Total volunteers vaccinated with mRNA-1273 and BNT126b2 vaccines and subgroups.

	mRNA-1273	BNT162b2
**Number of volunteers**	783	4187
1) Wane in IgG anti-RBD levels between 1st and 2nd extractions	767 (97.95%)	4068 (97.15%)
2) Increase in IgG anti-RBD levels between 1st and 2nd extractions	2 (0.25%)	51 (1.21%)
3) Increase in IgG anti-RBD between 1st and 2nd extraction (> 5,680 BAU/ml)	8 (1.02%)	45 (1.07%)
4) No detection of IgG anti-RBD on both extractions	1 (0.12%)	1 (0.02%)
5) IgG anti-RBD levels over the limit of quantification on 1st and 2nd extractions	5 (0.63%)	22 (0.52%)

Total number of volunteers for each vaccine. With respect to antibody levels, five categories were observed. Most volunteers presented a wane between both extractions. Some volunteers presented an increase in antibody levels between both extractions, and some reached values over the quantification limit. Other volunteers presented levels of antibodies over the higher limit of quantification at both extractions. Finally, one volunteer in each group did not present detectable antibodies at both extractions. For each group, the percentage indicates the frequency out of the total vaccinated volunteers for each vaccine group.

Among these volunteers, we observed five different patterns pertaining to their IgG anti-RBD levels (**Table 1**) (1): Most volunteers presented a wane in antibody levels from the first extraction to the second extraction (97.95% of the individuals vaccinated with mRNA-1273 and 97.15% vaccinated with BNT162b2) (2). However, some individuals presented an increase in antibody levels including 2 individuals vaccinated with mRNA-1273 (0.25% of mRNA-1273-vaccinated individuals) and 51 individuals vaccinated with BNT162b2 (1.21%) (3). Similarly, 8 individuals vaccinated with mRNA-1273 (1.02%) and 45 individuals vaccinated with BNT162b2 (1.07%) presented an increase in antibody levels, exceeding the higher limit of quantification (> 5,680 BAU/ml) at the second extraction. One explanation for this is that these individuals could have been infected between both extractions, since some had reported a positive SARS-CoV-2 test. However, because not all of them had a positive test, we could not conclude that all those individuals had been infected previously (4). The non-detection of antibodies occurred in only one individual in each group at the time of both extractions (0.12% and 0.02% in mRNA-1273 and BNT162b2 groups, respectively) (5). Finally, 5 individuals vaccinated with mRNA-1273 (0.63%) and 22 vaccinated with BNT162b2 (0.52%) presented antibody levels above the upper limit of quantification at both extractions.

Because most of the volunteers presented waning antibody levels, we focused our study on those individuals; detailed information about their characteristics is listed in **Table 2**. We found that 10 individuals from the 4,068 vaccinated with BNT162b2 had undetectable IgG anti-RBD levels on day 240, and they were removed from the study. In addition, 260 volunteers in the BNT162b2 group and 60 in the mRNA-1273 group were infected prior to vaccination, accounting for 6–7% of total volunteers. We decided to exclude those volunteers from the analysis. Therefore, the total number of individuals without a known COVID-19 history was 3,798 for the BNT162b2 cohort and 707 for the mRNA-1273 cohort. The number of volunteers, gender, mean age, and age ranges (number of individuals per range) were recorded. We also noted the mean days between the second dose and the first and second extractions. With respect to gender (*p*-value = 0.045) and age (*p*-value = 0.005), significant differences were observed between the vaccine groups. However, these differences were likely observed because of the large number of individuals and may not represent biological relevance between the vaccine cohorts. In terms of age, we decided to stratify the volunteers by age range, and no significant differences were observed (*p*-values > 0.05). Regarding the days of quantification, to simplify, we decided to name the first extraction as “Day 30” and the second as “Day 240” post-vaccination. For the first extraction, the mean number of days in the BNT162b2 group was 30.65 and in the mRNA-1273 group was 26.89 (*p*-value < 0.0001). In the second extraction, “Day 240” was 247.98 days in the BNT162b2 group and 237.19 days in the mRNA-1273 group (*p*-value < 0.0001). We assumed that despite the significant differences, a difference of 3 days over 30 days for the first extraction or a difference of 10 days over 240 days of follow-up would not alter the results.

**Table 2:** Characteristics of volunteers who exhibited a wane between the first and second extraction.

Characteristics	BNT162b2	mRNA-1273	p-value
Number of volunteers	3798	707
Gender (%)			0.045
Men	648 (17.06)	99 (14.00)	
Women	3150 (82.94)	608 (86.00)	
Age (years), mean (SD)	47.94 (11.25)	49.24 (10.84)	0.005
Age range 20 – 29 (%)	360 (9.48)	54 (7.64)	0.050
Age range 30 – 39 (%)	545 (14.35)	89 (12.59)	0.823
Age range 40 – 49 (%)	870 (22.90)	148 (20.93)	0.716
Age range 50 – 59 (%)	1421 (37.41)	284 (40.17)	0.349
Age range 60 – 69 (%)	602 (15.85)	132 (18.67)	0.191
Mean (SD) days between vaccination and first IgG anti-RBD quantification	30.65 (1.72)	26.89 (1.21)	<0.0001
Mean (SD) days between vaccination and second IgG anti-RBD quantification	247.98 (7.16)	237.19 (7.25)	<0.0001
Breakthroughs after vaccination, (%)	39 (1.03)	2 (0.28)	0.056
**Comorbidities**			
Number of volunteers	194	201	
Gender (Male/Female)	97/97	99/102	0.882
Age (years), mean (SD)	48.47 (11.22)	48.55 (10.64)	0.947
Presence of comorbidities			0.575
0 comorbidities	142	138	0.321
1 comorbidity	39	49	0.307
> 1 comorbidity	13	14	0.917
Type of comorbidity			
Cardiovascular history	23	22	0.776
Dyslipidaemia	15	10	0.261
Respiratory disease	8	11	0.531
Others	18	34	0.025

From the total volunteers, 260 vaccinated with BNT162b2 and 60 vaccinated with mRNA-1273 were discarded because they were infected before vaccination. The number of volunteers as well as gender, mean age, age ranges, and mean days between vaccination and first and second extractions are detailed for mRNA-1273- and BNT162b2-vaccinated volunteers. A two-sample t-test was used for the comparison of age and a chi-squared test was used for gender. To simplify, we decided to name the first extraction as “Day 30” and the second extraction as “Day 240”. Breakthrough infections until 1^st^ of December 2021 were noted for each vaccine cohort. A chi-squared test was used for the analysis. Finally, a randomized cohort of individuals vaccinated with BNT162b2 or mRNA-1273 was selected to study their comorbidities. The mean age was similar to that observed for total number of individuals. The presence of comorbidities was categorized as zero comorbidities, one comorbidity, or more than one comorbidity, indicating individuals with two or more comorbidities. Within comorbidities, the three major comorbidities were cardiovascular history, dyslipidaemia, and respiratory disease. A two-sample t-test was used for the comparison of age and a chi-squared test was used for the remaining variables.

**Table 2** shows details about breakthrough infections after vaccination, from the day of administration of the second dose until the 1^st^ of December 2021, when a third vaccination with a booster dose was offered to the healthcare worker in the center. During this period, 39 volunteers vaccinated with BNT162b2 were infected post-vaccination (1.03%), while 2 vaccinated with mRNA-1273 were infected (0.28%). Despite no significant differences (*p*-value = 0.056), it was interesting to note that four times more infections were registered in the BNT162b2 group than in the mRNA-1273 group.

Finally, because previous comorbidities may be a variable that influences IgG anti-RBD levels, we studied the presence of comorbidities in a smaller randomized cohort of volunteers. We searched for comorbidities in 194 individuals vaccinated with BNT162b2 and 201 vaccinated with mRNA-1273, with no differences between gender distribution and age (*p*-value > 0.05). We classified individuals into three groups: without comorbidities, presenting one comorbidity, or with more than one comorbidity. No significant differences were observed in any group (**Table 2**). We also subclassified comorbidities into cardiovascular history, dyslipidemia, and respiratory disease, and no differences were observed, except in the group with other types of comorbidities (including cancer, diabetes, and hypothyroidism), with those vaccinated with mRNA-1273 having the highest number of comorbidities (*p*-value = 0.025).

After comparison, we concluded that he differences in IgG anti-RBD levels could be attributable to the vaccine type since both cohorts were broadly similar.

### IgG anti-RBD levels with respect to vaccine types and individual factors

**Figure 1:**
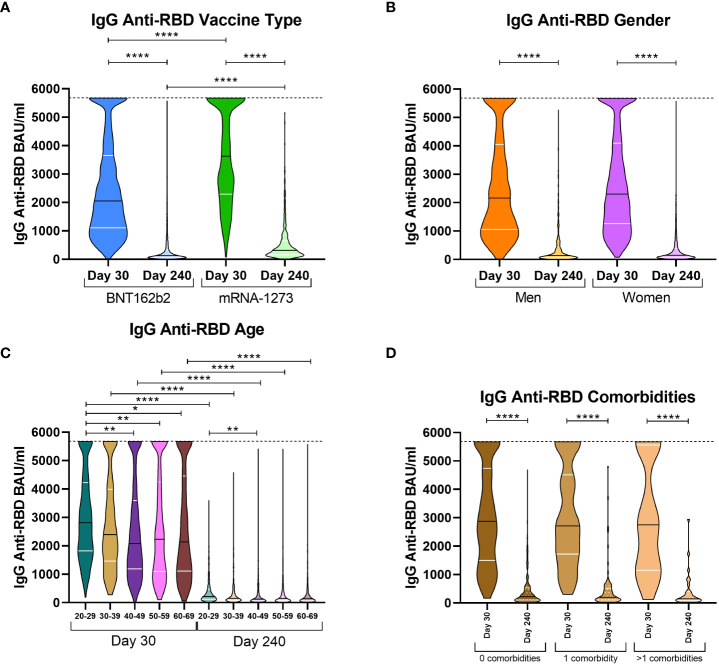
IgG anti-RBD levels on day 30 and 240 stratified by vaccine type, gender, age, and comorbidities. Violin plots of IgG anti-RBD levels in BAU/ml stratified according to vaccine type [**(A)** mRNA-1273 or BNT162b2]; gender [**(B)** male or female]; age ranges [**(C)** from 20 to 29 years old, 30-39 years, 40-49 years, 50-59 years, and 60-69 years]; and the presence of comorbidities [**(D)** no comorbidities, one comorbidity, or more than one comorbidity]. The median (black line) and quartiles (white lines) are represented in each violin plot. IgG anti-RBD levels were calculated in international units (BAU/ml). On day 30, the dotted line at 5,680 BAU/ml indicates the higher limit of quantification. A Kruskal Wallis test and a Dunn’s multiple comparisons test were performed for each variable. * p-value < 0.05, ** p-value < 0.01, **** p-value < 0.0001.

We aimed to compare antibody levels on both extraction days and the influence of the type of vaccine, gender, age, and comorbidities on the immunoglobulin response of those volunteers who exhibited a wane in IgG anti-RBD levels, since they constituted the majority of volunteers in this study. Comparing volunteers by type of vaccine, significant differences were observed on both days 30 and 240. On day 30, BNT162b2 volunteers presented with a median of 2,053.47 BAU/ml while mRNA-1273 volunteers presented with 3,625.70 BAU/ml (**Figure 1A**). On day 240, antibody levels decreased abruptly for both types of vaccines; however, mRNA-1273 volunteers still had higher levels (312.14 vs. 126.47 for mRNA-1273 and BNT162b2, respectively). With respect to the implication of gender in COVID-19 severity (Statsenko et al., 2021), we decided to stratify volunteers by their gender to study if this variable impacted anti-SARS-CoV-2 immune memory. Despite the wane in antibody levels between both extractions, there were no differences between men and women on days 30 and 240 (**Figure 1B**). We also studied the effect of age on immune memory. We decided to group volunteers according to age in 10-year intervals (from 20 to 29 years old, 30-39 years, 40-49 years, 50-59 years, and 60-69 years). We observed that on day 30, the median IgG anti-RBD levels in the 20-29 age group were higher than in the rest of the groups (2,812.60 vs. 2,395.36 (30-39 years, p > 0.999); 2,083.48 (40-49 years, p = 0.0039); 2,231.22 (50-59 years, p = 0.0091); and 2,2135.47 (60-69 years, p = 0.0212), **Figure 1C**). However, on day 240, part of these differences was lost, and levels were only higher in the 20-29 age group compared to the 40-49 group (213.93 vs. 120.75, respectively, p = 0.0019). Finally, we studied IgG anti-RBD levels in the randomized cohort of 395 volunteers, classified by individuals without comorbidities, those with the presence of one comorbidity, or those with more than one comorbidity (**Figure 1D**). In this case, the three groups were very similar, both at day 30 and day 240; thus, the comorbidities were not associated with an increase in the waning of IgG levels.

With respect to these results, it seemed that mRNA-1273 presented higher IgG levels at both extraction days and likely could better protect from SARS-CoV-2 infection than BNT162b2. In addition, we observed that age was also implicated in the humoral response; as expected, the younger individuals produced higher levels of antibodies. For gender, despite the fact that no differences were observed in the total sample, it could have implications associated with age and the type of vaccine administered, as we previously observed for the cellular response (Gil-Manso et al., 2022).

### Individuals stratified by their characteristics identified different behaviour between mRNA vaccines

**Figure 2:**
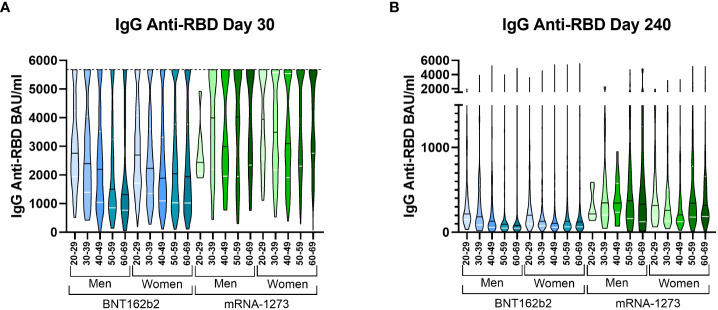
IgG anti-RBD levels on day 30 and 240 stratified by all three variables simultaneously. Violin plots representing the IgG anti-RBD levels in BAU/ml stratified by vaccine type, gender, and age on day 30 **(A)** and day 240 **(B)**. The median (black line) and quartiles (white lines) are represented in each violin plot. On day 30, the dotted line at 5,680 BAU/ml indicates the higher limit of quantification. A Kruskal Wallis test and a Dunn’s multiple comparisons test were performed for each variable. IgG anti-RBD levels were calculated in international units (BAU/ml).

We then decided to simultaneously stratify the volunteers based on the type of vaccine received, age, and gender. On day 30, we observed that the median IgG level in volunteers vaccinated with mRNA-1273 was higher than in any other group of volunteers vaccinated with BNT162b2 (**Figure 2A**). We observed a clear and distinct pattern between the vaccines with age; volunteers vaccinated with BNT162b2 presented higher antibody levels in the younger groups, while those levels were partially lost in the older volunteers, especially in men. However, the opposite pattern was observed in the mRNA-1273 group, where older individuals presented higher antibody levels, although the difference was not significant. On day 240, almost all mRNA-1273 groups still had higher levels. The pattern observed on day 30 in the IgG anti-RBD levels of the mRNA-1273 group was still conserved on day 240, but the differences between ages were reduced. Meanwhile, the pattern observed in the BNT162b2 volunteers on day 30 was conserved on day 240 (**Figure 2B**). The significant differences between each group are detailed in **Supplemental Figure S1**.

**Figure 3:**
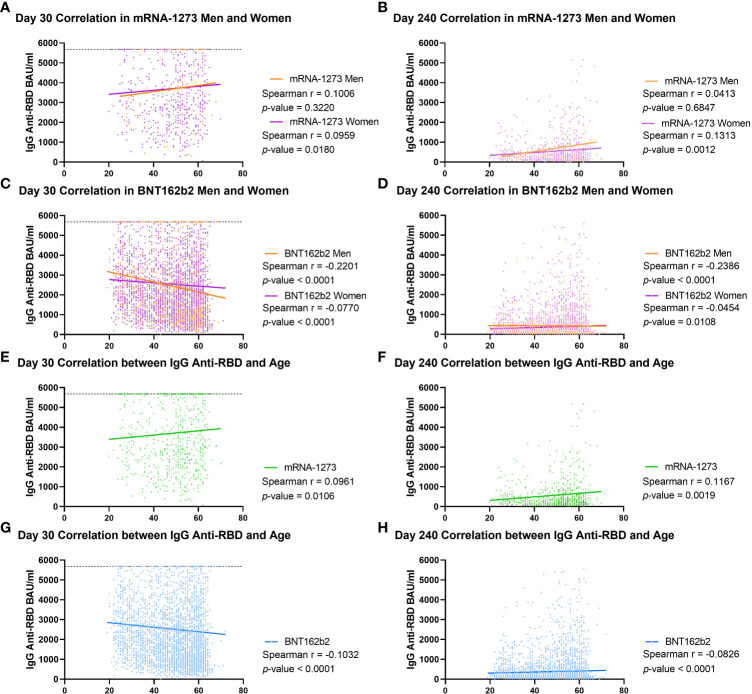
Correlation between IgG anti-RBD levels and age in cohorts stratified by type of vaccine and gender. Spearman correlation was calculated for male and female volunteers vaccinated with mRNA-1273 on day 30 **(A)** and 240 **(B)** and for men and women vaccinated with BNT162b2 on day 30 **(C)** and 240 **(D)**. This analysis was performed in all volunteers, regardless of gender, vaccinated with mRNA-1273 on day 30 **(E)** and day 240 **(F)** or with BNT162b2 [day 30 **(G)**, and day 240 **(H)**]. IgG anti-RBD levels were calculated as in international units (BAU/ml). Each dot indicates an individual value, and the lines indicate linear regression. On day 30, the dotted line at 5,680 BAU/ml indicates the higher limit of quantification. The Spearman r and *p*-value are indicated for each group. *p*-values < 0.05 were considered significant.

These findings were supported by the calculation of the correlation between age and the antibody values in each group classified by gender and vaccine type (**Figure 3**). In the mRNA-1273 group, we obtained a Spearman r of 0.1006 (p = 0.3220) for men and r of 0.0959 (p = 0.0180) for women on day 30, increasing on day 240 for women (r = 0.1313; p = 0.0012) but not for men (r = 0.0413; p = 0.6847, **Figures 3A, B**). In the case of the BNT162b2 group, on day 30, both men and women had a negative Spearman correlation (r = -0.2201; p < 0.0001 and r = -0.0770; p < 0.0001, respectively, **Figure 3C**), and this trend was maintained on day 240, with r = -0.2386 (p < 0.0001) for men and r = -0.0454 (p = 0.0108) for women (**Figure 3D**). When these correlations were performed without stratifying by gender, similar results were obtained, with the mRNA-1273 Spearman r on day 30 equal to 0.0961 (*p*-value = 0.0106) and increasing to r = 0.1167 (*p*-value = 0.0019) on day 240 (**Figures 3E, F**). However, in the case of the BNT162b2 group, this correlation remained negative both on day 30 (r = -0.1032; *p*-value < 0.0001) and on day 240 (r = -0.0826; *p*-value < 0.0001, **Figures 3G, H**).

Altogether, these results indicate that vaccination with mRNA-1273 conferred higher IgG anti-RBD levels than BNT162b2 during follow-up. They also revealed that age is a key factor, depending on the vaccine administered.

### mRNA-1273 induced higher IgG Anti-RBD levels during follow-up

**Figure 4:**
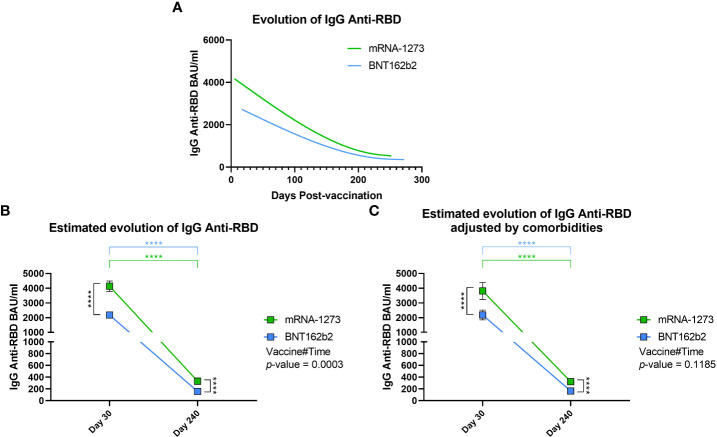
Evolution of IgG Anti-RBD levels during follow-up. **(A)** Spline representation of the evolution of IgG anti-RBD levels in both mRNA-1273- and BNT162b2-vaccinated individuals. For each individual, the exact day post-vaccination was selected. The spline representation was made by smoothing the spline with three knots. **(B)** Estimated evolution of IgG anti-RBD levels for both vaccines adjusted by age and gender, calculated using a mixed-effects model with transformed logarithmic IgG anti-RBD values and with tobit regression. Vaccine#Time indicates the behaviour of each vaccine during the follow-up. Squares indicate the estimated mean, and brackets indicate a 95% confidence interval. **(C)** In the randomized cohort, the estimated evolution of IgG anti-RBD levels for both vaccines was adjusted by age, gender, and comorbidities in a multivariable analysis. **** *p*-value < 0.0001.

After the previous analyses comparing differences between groups on day 30 and day 240, we decided to study the evolution of IgG anti-RBD levels during the follow-up. First, we made a spline interpolation of the levels of IgG anti-RBD considering the exact day on which the sample was taken. From this, we observed that the IgG anti-RBD levels in mRNA-1273-vaccinated volunteers were higher from the beginning and remained above that observed in BNT162b2-vaccinated volunteers throughout the follow-up (**Figure 4A**). This finding was supported when we analyzed IgG anti-RBD levels using a mixed-effects model adjusted by age and gender, which was used to calculate the estimated mean for both vaccines on days 30 and 240, the evolution between time points, and the interaction between vaccines during the follow-up. We observed that, as we have seen previously, the estimated mean of IgG anti-RBD levels in mRNA-1273-vaccinated volunteers was higher than in the BNT162b2 group on day 30 (p < 0.0001) and day 240 (p < 0.0001, **Figure 4B**). In addition, we calculated that the decrease in IgG anti-RBD levels was about 10-fold during the follow-up for both vaccines (p < 0.0001). Finally, we observed that the evolution of both vaccines was different since the mixed-effects model presented a Vaccine#Time *p*-value equal to 0.0003. In this case, the evolution of IgG anti-RBD levels in mRNA-1273-vaccinated volunteers showed a greater decrease. When analyzing evolution considering comorbidities, the Vaccine#Time value was not significant (*p*-value = 0.1185), with both vaccines having a similar evolution during time (**Figure 4C**).

However, despite the adjustment by comorbidities, **Figure 4**–comparing both vaccines–shows a very similar trend, highlighting mRNA-1273 as the vaccine that conferred the highest levels of IgG anti-RBD during the entire follow-up.

**Figure 5:**
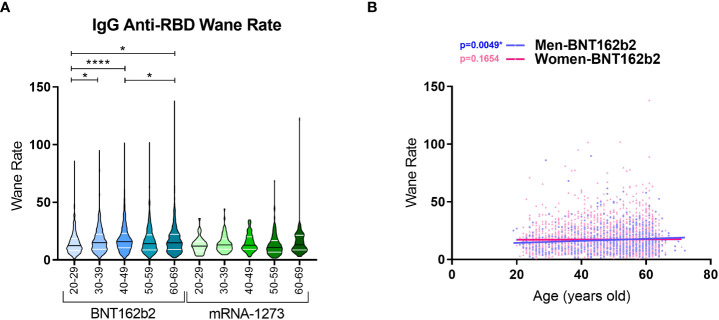
Wane ratios of IgG anti-RBD levels in BNT162b2- and mRNA-1273-vaccinated individuals with respect to age ranges. **(A)** Violin plots represent the waning rate of IgG anti-RBD levels for each individual. The wane rate was calculated by dividing the IgG anti-RBD level on day 30 by the IgG Anti-RBD level on day 240 for each individual. The median (black line) and quartiles (white lines) are represented in the violin plots. A Kruskal Wallis test and a Dunn’s multiple comparisons test were performed for each variable. **(B)** Spearman correlation was calculated for male and female volunteers vaccinated with BNT162b2. * *p*-value < 0.05, **** *p*-value < 0.0001.

Finally, we calculated the wane ratio of the IgG anti-RBD levels between day 30 and 240 for each vaccine and age range. We found that the median wane ratio in the BNT162b2 cohort was higher than that in the mRNA-1273 cohort for all age ranges, including 20-29 years (12.41 vs. 12.02), 30-39 years (15.06 vs. 13.00), 40-49 years (15.90 vs. 12.82), 50-59 years (14.16 vs. 10.91), and 60-69 years (14.92 vs. 13.15 for BNT162b2 vs. mRNA-1273, respectively; **Figure 5A**). In the BNT162b2 cohort, differences were found regarding age, with older individuals presenting a higher ratio of wane than the younger volunteers. In the mRNA-1273 cohort, the wane ratio was similar and comparable between age ranges. We then analysed the waning rate in the BNT162b2 cohort with respect to gender. Interestingly, a significant positive correlation between age and the waning rate was observed in men (*p*-value = 0.0049) but not women (*p*-value = 0.1654) (**Figure 5B**).

These results show that IgG anti-RBD levels decreased drastically during the follow-up for both vaccines, with the mRNA-1273 vaccine inducing higher levels over the eight-month follow-up. Moreover, in terms of the wane rates in every age range, BNT162b2 showed a higher wane rate in general; this was especially prevalent in older individuals, where men presented the highest decrease.

## Discussion

The development of mRNA-based vaccines was a major breakthrough in the attempt to halt the advance of the COVID-19 pandemic. Both mRNA-based vaccines discussed here were developed faster than ever and distributed worldwide (Pilkington et al., 2022). They have been proven to be safe and capable of inducing a fast humoral and cellular post-vaccination response, followed by the production of anti-S, anti-RBD, or neutralizing antibodies and cellular cytokines after SARS-CoV-2-derived peptide pool stimulation, reaching a maximum peak of both responses in the first 4-6 weeks after the second dose (Gil-Manso et al., 2021; Goel et al., 2021; Keshavarz et al., 2022). Subsequently, the specific anti-SARS-CoV-2 responses fade over the months, reaching minimum but detectable levels at 6-9 months (Doria-Rose et al., 2021; Naaber et al., 2021; Herzberg et al., 2022; Jalkanen et al., 2022; Keshavarz et al., 2022). Here, we observed that our results aligned with what has already been published, since we observed high levels of anti-RBD antibodies on day 30 and a drastic reduction over 8 months post-vaccination, although still detectable. Moreover, comparing the evolution between vaccines, we found that during all follow-ups, IgG anti-RBD levels induced by the mRNA-1273 vaccine remained above those induced by the BNT162b2 vaccine. Wei et al. demonstrated that a higher level of approximately 100 BAU/ml caused by BNT162b2 vaccine could be protective against infection (Wei et al., 2022). No information was found on the mRNA1273 vaccine. At day 240 post-vaccination, 41% of the individuals vaccinated with BNT162b2 presented levels of IgG anti-RBD inferior to this value. This could explain why during the follow-up, there were four times more vaccine breakthroughs in BNT162b2-vaccinated individuals than in the mRNA1273 cohort. These results are in line with data on the efficacy of the vaccines against symptomatic infection and severity, which suggest the mRNA-1273 vaccine has improved effectiveness when compared to BNT162b2 (Tang et al., 2021; Dickerman et al., 2022). In addition, the wane in the humoral and cellular responses coincided with the apparition of SARS-CoV-2 variants (Fabiani et al., 2021; Lin et al., 2022). Moreover, the emergence of new variants has led to an increase in infections, which has subsequently resulted in the administration of a third booster dose in many countries. The third dose, especially a heterogeneous vaccination, induced a boost in the humoral and cellular anti-SARS-CoV-2 responses (Munro et al., 2021) and was more effective against the new variants than only two doses (Tartof et al., 2022; Thompson et al., 2022). However, the administration of this third dose is controversial, since three doses in less than one year could cause the hyperstimulation of the immune system, which could be detrimental to immune memory by inducing cellular exhaustion, as has been described for repeated influenza vaccinations inducing declining protection (Khurana et al., 2019; Sugishita et al., 2020).

We also found that the mRNA-1273 vaccine induced higher IgG anti-RBD levels than BNT162b2. This difference has already been described (Wheeler et al., 2021; Keshavarz et al., 2022); however, with our large number of volunteers, we corroborated that the differences between responses to both vaccines were clear. The fact that the mRNA-1273 vaccine generates an enhanced humoral response could be related to its higher efficiency compared to BNT162b2 (Lin et al., 2022; Premikha et al., 2022). In addition, in individuals on dialysis (Kaiser et al., 2021; Yau et al., 2022) or in immunocompromised patients (Obeid et al., 2022), it has been observed that the mRNA-1273 vaccine induces a higher humoral response, which would support the use of this vaccine in vulnerable populations. In addition, we observed that older individuals vaccinated with BNT162b2 had lower antibody levels (< 70 years old), as has already been reported (Naaber et al., 2021; Bai et al., 2022; Herzberg et al., 2022). To the contrary, as far as we know, we described for the first time individuals vaccinated with mRNA-1273 who presented the opposite behaviour: older volunteers (< 70 years old) appeared to present with a higher humoral response than younger participants. However, the main limitation of this study is that the healthcare workers recruited were under 70 years old; thus, we cannot extend this conclusion to older populations (> 70 years old). Nevertheless, positive correlations between IgG anti-RBD levels and age for mRNA-1273-vaccinated individuals may suggest that individuals over 70 could have higher IgG anti-RBD levels. Furthermore, individuals vaccinated with BNT126b2 presented a negative correlation between IgG anti-RBD levels and age, and older individuals vaccinated with BNT162b2 had the highest wane ratios, especially men vaccinated with BNT162b2. This evidence may further support the use of the mRNA-1273 vaccine not only in at-risk populations, such as healthcare workers, but also in the elderly population, as it may achieve higher IgG anti-RBD levels than the BNT162b2 vaccine and, in line with previous publications, an enhanced anti-SARS-CoV-2 cellular response (Gil-Manso et al., 2022).

These differences between vaccines may be due to the difference between the number of days separating the administration of the two doses (21 vs. 28 days for BN162b2 vs. mRNA-1273, respectively [Jackson et al., 2020; Polack et al., 2020)] or their different lipidic composition (Schoenmaker et al., 2021), which could be responsible for their different behaviour with respect to the immune memory response. Another potential reason is that each injection of BNT162b2 includes 0.3 mL containing 30 µg of mRNA [concentration of 100 µg/mL (Polack et al., 2020)]. In comparison, the infusion of mRNA-1273 has three times more mRNA [0.5 mL per injection, containing 100 µg (Baden et al., 2021)]. For this reason, further studies should attempt to better understand the mechanisms of each vaccine in the host.

We are aware of the limitations of this study, for example, the quantification being restricted to anti-RBD antibodies. It would have been interesting to quantify neutralizing antibodies, conduct a B cell analysis, or determine the avidity of the antibodies, which could provide more robust results about humoral memory. In addition, we did not include other anti-SARS-CoV-2 vaccines, and we did not continue the follow-up past 240 days; therefore, we did not perform serology measurements after the third booster dose of the mRNA-based vaccines. Further studies on the effect of the third dose and the combination of vaccines could provide more information about protection and the most ideal vaccine regimen. Moreover, information about the cellular response and immune cell populations could provide a vast spectrum of knowledge of how the immune system evolves after anti-SARS-CoV-2 vaccination.

In conclusion, mRNA-based vaccines have proven to be a rapidly engineered tool for conferring immunity globally. Comparing the two most prominent mRNA vaccines available in Spain, we found that the mRNA-1273 vaccine achieved higher levels of anti-RBD antibodies when compared to BNT162b2 after two doses, where age may be a highly implicated factor. These results could be used to develop vaccination strategies to achieve improved and long-lasting anti-SARS-CoV-2 immunity in at-risk populations.

## Gregorio Marañón Microbiology-ID COVID-19 study group members

Luis Alcalá, Teresa Aldámiz, Roberto Alonso, Beatriz Álvarez, Ana Álvarez-Uría, Alexi Arias, Elena Bermúdez, Emilio Bouza, Sergio Buenesta do-Serrano, Almudena Burillo, Raquel Carrillo, Pilar Catalán, Emilia Cercenado, Alejandro Cobos, Cristina Díez, Pilar Escribano, Agustín Estévez, Chiara Fanciulli, Alicia Galar, Mª Dolores García, Darío García de Viedma, Paloma Gijón, Adolfo González, Helmuth Guillén, Jesús Guinea, Marta Herranz, Álvaro Irigoyen, Laura Vanessa Haces, Martha Kestler, Juan Carlos López, Carmen Narcisa Losada, Marina Machado, Mercedes Marín, Pablo Martín-Rabadán, Andrea Molero-Salinas, Pedro Montilla, Patricia Muñoz, María Olmedo, Belén Padilla, Rosalía Palomino-Cabrera, María Palomo, María Jesús Pérez-Granda, Daniel Peñas-Utrilla, Laura Pérez-Lago, Leire Pérez, Elena Reigadas, Cristina Rincón, Belén Rodríguez, Sara Rodríguez, Cristina Rodríguez-Grande, Adriana Rojas, María Jesús Ruiz-Serrano, Carlos Sánchez, Mar Sánchez, Amadeo Sanz-Pérez, Julia Serrano, Francisco Tejerina, Maricela Valerio, Mª Cristina Veintimilla, Lara Vesperinas,Teresa Vicente, Sofíade la Villa

## Data availability statement

The original contributions presented in the study are included in the article/**Supplementary materials**. Further inquiries can be directed to the corresponding authors.

## Ethics statement

The studies involving human participants were reviewed and approved by Ethics Committee of the Hospital General Universitario Gregorio Marañón (MICRO.HGUGM.2020-021). The patients/participants provided their written informed consent to participate in this study.

## Author contributions

Conceptualization of the study: RA, PC, RC-R, and PM. Coordination and obtainment of biological samples, and the conduction of the demographic and epidemiological survey: IS-A, and MM. Review of clinical records: SG-M and RA. Implementation of experiments: RA and PC. Acquisition, analysis, and interpretation of data: SG-M, RA, and MP. Discussion of results: SG-M, RA, RC-R, MP, and RM. Draft of the original work: SG-M, RA, and MP All authors approved the submitted version of the manuscript. The authors declare no competing financial interest.

## Funding

SG-M was supported by the Youth Employment Program, co-financed by the Madrid community, and FEDER Founds (PEJ-2020-AI/BMD-17954). The funders had no role in the study design, data collection, data analysis, decision to publish, or manuscript preparation. This work was partially supported by grants from the Carlos III Health Research Institute (ISCIII) (PI18/00506), co-funded by ERDF (FEDER) Funds from the European Commission, “A way of making Europe”, and from the ACT4COVID consortium (CellNex funding).

## Acknowledgments

The authors would like to thank all the health workers who participated in this study. We would also thank José María Bellón from the Biostatistics Unit of the IiSGM for his help.

## Conflict of interest

The authors declare that the research was conducted in the absence of any commercial or financial relationships that could be construed as a potential conflict of interest.

## Publisher’s note

All claims expressed in this article are solely those of the authors and do not necessarily represent those of their affiliated organizations, or those of the publisher, the editors and the reviewers. Any product that may be evaluated in this article, or claim that may be made by its manufacturer, is not guaranteed or endorsed by the publisher.
