# Supplementary material for: IgG anti-RBD levels during 8-month follow-up post-vaccination with BNT162b2 and mRNA-1273 vaccines in healthcare workers: A one-center study
Source: Front Cell Infect Microbiol. 2022 Nov 30;12:1035155. doi: 10.3389/fcimb.2022.1035155 (PMC9748346; doi:10.3389/fcimb.2022.1035155)
Supplement: Supplementary file 1 [file DataSheet_1.pdf]

frontiers

Supplementary Material

			BNT162b2										mRNA-1273									
			Male					Female					Male					Female				
		Years	20-29	30-39	40-49	50-59	60-69	20-29	30-39	40-49	50-59	60-69	20-29	30-39	40-49	50-59	60-69	20-29	30-39	40-49	50-59	60-69
BNT162b2	Male	20-29		>0.9999	>0.9999	0.0017	0.0013	>0.9999	>0.9999	0.0510	0.2532	0.1520	>0.9999	>0.9999	>0.9999	>0.9999	>0.9999	>0.9999	>0.9999	>0.9999	>0.9999	0.9974
		30-39	>0.9999		>0.9999	0.1245	0.0747	>0.9999	>0.9999	>0.9999	>0.9999	>0.9999	>0.9999	>0.9999	>0.9999	>0.9999	>0.9999	0.1289	>0.9999	>0.9999	0.0028	0.0006
		40-49	0.0502	>0.9999		>0.9999	>0.9999	0.4556	>0.9999	>0.9999	>0.9999	>0.9999	>0.9999	>0.9999	>0.9999	0.0307	0.2700	0.0006	0.0056	0.0036	<0.0001	<0.0001
		50-59	<0.0001	0.0029	>0.9999		>0.9999	<0.0001	0.0121	>0.9999	0.5813	>0.9999	>0.9999	0.1331	0.2497	<0.0001	0.0047	<0.0001	<0.0001	<0.0001	<0.0001	<0.0001
		60-69	<0.0001	0.0033	>0.9999	>0.9999		<0.0001	0.0236	>0.9999	0.5658	>0.9999	>0.9999	0.0689	0.1263	<0.0001	0.0024	<0.0001	<0.0001	<0.0001	<0.0001	<0.0001
	Female	20-29	>0.9999	>0.9999	0.1396	<0.0001	<0.0001		>0.9999	<0.0001	0.0002	0.0003	>0.9999	>0.9999	>0.9999	>0.9999	>0.9999	0.4567	>0.9999	>0.9999	0.0012	0.0006
		30-39	0.0240	>0.9999	>0.9999	0.3822	0.4223	0.0086		0.7043	>0.9999	>0.9999	>0.9999	>0.9999	>0.9999	0.0944	0.7058	0.0015	0.0139	0.0053	<0.0001	<0.0001
		40-49	<0.0001	0.0059	>0.9999	>0.9999	>0.9999	<0.0001	0.6092		>0.9999	>0.9999	>0.9999	0.7223	>0.9999	0.0007	0.0450	<0.0001	<0.0001	<0.0001	<0.0001	<0.0001
		50-59	0.0096	>0.9999	>0.9999	0.1378	0.2580	0.0003	>0.9999	0.0556		>0.9999	>0.9999	>0.9999	>0.9999	0.0035	0.1177	<0.0001	<0.0001	<0.0001	<0.0001	<0.0001
		60-69	0.0029	0.5080	>0.9999	>0.9999	>0.9999	0.0001	>0.9999	>0.9999	>0.9999		>0.9999	>0.9999	>0.9999	0.0021	0.0789	>0.9999	>0.9999	>0.9999	>0.9999	>0.9999
mRNA-1273	Male	20-29	>0.9999	>0.9999	>0.9999	>0.9999	>0.9999	>0.9999	>0.9999	>0.9999	>0.9999	>0.9999		>0.9999	>0.9999	>0.9999	>0.9999	>0.9999	>0.9999	>0.9999	>0.9999	>0.9999
		30-39	>0.9999	>0.9999	>0.9999	0.0726	0.0413	>0.9999	>0.9999	0.1553	>0.9999	0.6959	>0.9999		>0.9999	>0.9999	>0.9999	>0.9999	>0.9999	>0.9999	>0.9999	>0.9999
		40-49	>0.9999	>0.9999	>0.9999	0.0432	0.0242	>0.9999	>0.9999	0.0995	>0.9999	0.5707	>0.9999	>0.9999		>0.9999	>0.9999	>0.9999	>0.9999	>0.9999	>0.9999	>0.9999
		50-59	>0.9999	>0.9999	0.0020	<0.0001	<0.0001	>0.9999	0.0010	<0.0001	0.0004	0.0001	>0.9999	>0.9999	>0.9999		>0.9999	>0.9999	>0.9999	>0.9999	>0.9999	>0.9999
		60-69	>0.9999	>0.9999	>0.9999	0.0613	0.0343	>0.9999	>0.9999	0.1414	>0.9999	0.8083	>0.9999	>0.9999	>0.9999	>0.9999		>0.9999	>0.9999	>0.9999	>0.9999	>0.9999
	Female	20-29	>0.9999	>0.9999	0.0014	<0.0001	<0.0001	>0.9999	0.0006	<0.0001	0.0002	<0.0001	>0.9999	>0.9999	>0.9999	>0.9999	>0.9999		>0.9999	>0.9999	>0.9999	>0.9999
		30-39	>0.9999	>0.9999	0.0118	<0.0001	<0.0001	>0.9999	0.0036	<0.0001	0.0011	0.0003	>0.9999	>0.9999	>0.9999	>0.9999	>0.9999	>0.9999		>0.9999	>0.9999	>0.9999
		40-49	>0.9999	>0.9999	0.0187	<0.0001	<0.0001	>0.9999	0.0025	<0.0001	0.0003	<0.0001	>0.9999	>0.9999	>0.9999	>0.9999	>0.9999	>0.9999	>0.9999		>0.9999	>0.9999
		50-59	>0.9999	0.0314	<0.0001	<0.0001	<0.0001	0.0004	<0.0001	<0.0001	<0.0001	<0.0001	>0.9999	>0.9999	>0.9999	>0.9999	>0.9999	>0.9999	>0.9999	>0.9999		>0.9999
		60-69	>0.9999	0.2223	<0.0001	<0.0001	<0.0001	0.0410	<0.0001	<0.0001	<0.0001	<0.0001	>0.9999	>0.9999	>0.9999	>0.9999	>0.9999	>0.9999	>0.9999	>0.9999	>0.9999	

Day 30

Day 240

Supplemental Figure 1. Significant differences between groups classified by vaccine type, age, and gender. *p*-values for all comparisons after a Kruskal Wallis test and Dunn's multiple comparisons test are indicated in comparisons on day 30 (top right-hand side) and day 240 (bottom left-hand side). The four shades of orange, from light to dark, indicate the significance levels $p < 0.05$, $p < 0.01$, $p < 0.001$, and $p < 0.0001$, respectively.
